# A Multidimensional Approach for Evaluating Reality in Social Media: Mixed Methods Study

**DOI:** 10.2196/52058

**Published:** 2024-08-06

**Authors:** HyunYi Cho, Wenbo Li, Rachel Lopez

**Affiliations:** 1 The Ohio State University Columbus, OH United States; 2 Stony Brook University Stony Brook, NY United States

**Keywords:** fake, fact, misinformation, reality, social media, scale, measure, instrument, user-centric, tailoring, digital media literacy

## Abstract

**Background:**

Misinformation is a threat to public health. The effective countering of misinformation may require moving beyond the binary classification of fake versus fact to capture the range of schemas that users employ to evaluate social media content. A more comprehensive understanding of user evaluation schemas is necessary.

**Objective:**

The goal of this research was to advance the current understanding of user evaluations of social media information and to develop and validate a measurement instrument for assessing social media realism.

**Methods:**

This research involved a sequence of 2 studies. First, we used qualitative focus groups (n=48). Second, building on the first study, we surveyed a national sample of social media users (n=442). The focus group data were analyzed using the constant comparison approach. The survey data were analyzed using confirmatory factor analyses and ordinary least squares regression.

**Results:**

The findings showed that social media reality evaluation involves 5 dimensions: falsity, naturality, authenticity, resonance, and social assurance. These dimensions were differentially mapped onto patterns of social media use. Authenticity was strongly associated with the existing global measure of social media realism (*P*<.001). Naturality, or the willingness to accept artificiality and engineered aspects of social media representations, was linked to hedonic enjoyment (*P*<.001). Resonance predicted reflective thinking (*P*<.001), while social assurance was strongly related to addictive use (*P*<.001). Falsity, the general belief that much of what is on social media is not real, showed a positive association with both frequency (*P*<.001) and engagement with (*P*=.003) social media. These results provide preliminary validity data for a social media reality measure that encompasses multiple evaluation schemas for social media content.

**Conclusions:**

The identification of divergent schemas expands the current focus beyond fake versus fact, while the goals, contexts, and outcomes of social media use associated with these schemas can guide future digital media literacy efforts. Specifically, the social media reality measure can be used to develop tailored digital media literacy interventions for addressing diverse public health issues.

## Introduction

### Background

The surgeon general of the United States declared that misinformation is a serious threat to public health and urged all Americans to help deter its spread [1]. Research has found that misinformation is widespread in diverse domains of public health including, but not limited to, drugs and smoking, communicable and noncommunicable diseases, eating disorders, treatments and medical interventions, and mental health [2,3].

Given its ubiquity, addressing misinformation requires whole-of-society efforts [1]. At the same time, the surgeon general’s advisory calls on researchers and educators to take specific actions. Researchers are encouraged to develop a user-centric understanding of how different individuals interpret and interact with information on social media. On the basis of this understanding, educators are asked to offer programs that assist diverse individuals to become more discerning consumers of digital media content.

Overall, there seems to be a paradoxical reliance on, and distrust in, social media. Research suggests that users generally doubt the accuracy of social media information [4,5]; for example, a recent survey found that 62% of participants from 7 countries, including the United States, reported seeing misinformation on social media [4]. Another study found that across a variety of digital media contexts, participants consistently expected other users to lie about their appearance [5]. Concurrently, 72% of Americans use social media platforms and visit them at least once a day [6].

The extensive social media use and simultaneous concern about misinformation suggest that the motivations for, and gratifications from, social media use may go beyond obtaining factual information. Previous research in mass media has shown that user evaluations of realism use multiple dimensions [7,8], that is, users use divergent schemas or guiding frameworks comprising a collection and structure of information when interpreting media messages. Similarly, studies suggest that when evaluating social media information, users may use more than just a dichotomy of fake versus fact [9-11], and different evaluative schemas may be used for different goals and contexts [12,13]. However, despite this understanding, a comprehensive examination of these dimensions has yet to be undertaken.

### Objectives

The goal of this research was to advance the current knowledge about user evaluations of social media content and to develop and initially validate a measurement instrument for assessing social media realism. Given the pervasiveness of misinformation [1-3], our goal was to offer a general measure that can be used in diverse public health domains. These efforts aim to inform and enhance public health misinformation–countering efforts using digital media literacy. Toward this end, 2 studies were conducted. First, qualitative focus group research examined salient themes in social media realism judgments among users. Second, building on the identified themes and existing literature, a quantitative survey study investigated the schemas’ connections with patterns of social media use.

### Conceptual Foundation

User judgments of media’s correspondence with reality are commonly referred to as perceived realism. This construct has been found to be one of the primary determinants of media effects on various public health behaviors and outcomes. One of these domains is violence prevention. Research indicates that when viewers perceive harmful media such as violent content to be highly realistic, the effects are amplified [14,15]. By contrast, melanoma prevention interventions using a media literacy approach found that correcting perceived media realism related to tanned looks was central to the intervention efficacy evidenced at a 6-month follow-up [16,17]. Anti–substance abuse public service announcements were more persuasive when viewers judged that the realism of the messages was high rather than low [7].

Perceived realism has also been found to be important in determining social media effects pertaining to health behaviors; for instance, exposing individuals to a juxtaposition of real and idealized Instagram images of the same woman reduced body dissatisfaction compared to exposure to either the real image or ideal image alone [18]. Perceived realism moderated the effects of exposure to social media e-cigarette messages among teenagers. When perceived realism was low, the effect of the exposure on the provaping attitude was mitigated [19].

Moreover, research suggests that user judgments of social media information may be multifaceted. One of the facets is authenticity, which refers to the congruency between digital and offline selves and behaviors [20]. Authenticity has been a focus of studies on social media realism effects on health behaviors. Cho et al [19] investigated teenagers’ consideration of how user-created content on social media reflected the content creators’ true selves using items such as “People’s posts portray how they really live their lives.” Similarly, Tiggemann and Anderberg [18] assessed young women’s perceptions using items such as “The models in the advertisements looked like they would look like in person.” Importantly, these studies found that a low perception of authenticity reduced the harmful effects of social media on e-cigarette use and eating disorders [18,19]. Recognizing this significance, Jenkins et al [21] called for more public health research assessing the predictors of the perceptions of authenticity concerning nutrition messages on social media.

Authenticity may not be the only dimension users consider when evaluating social media content. Studies suggest that users may rely on social cues to judge the proximity of social media posts to reality. In addition to the core content of the message, the metrics generated by others may provide a bandwagon heuristic for assessing social media messages [22]. Popularity cues such as others’ support and approval of a message may provide a rule of thumb for evaluating the credibility of digital media [23]; for instance, the effects of both pro- and antivaping social media messages were increased by strong rather than weak popularity cues [24].

We suspect that another criterion people use to evaluate social media messages may be resonance. A significant body of research has documented that, on social media, users tend to prefer information that is consonant, rather than dissonant, with their existing beliefs. Communication on social media has often been described as occurring in “echo chambers” where users’ attitudes are reinforced through repeated interactions with like-minded others [25]. Related mechanisms include selective exposure [26] and confirmation bias [27], whereby people seek information consistent with their preexisting perspectives [28] and process information in a way that affirms their prior values [29]. This biased process suggests that users may give more credibility and evaluate more positively the social media messages that resonate with their predispositions. Exposure to opposing positions can facilitate polarization through heightened social identities on social media [30].

## Study 1: Qualitative Focus Groups

### Overview

Existing research suggests that users may be skeptical of misinformation on social media, but they may also consider other factors, such as authenticity, when evaluating the content, rather than relying on a binary distinction of fake versus fact. However, there is still a limited understanding of potential additional factors. To address this gap in knowledge, we used a qualitative method, focus groups, to identify these factors.

### Methods

#### Design and Participants

For our focus group research, we recruited 48 social media users from a large public university in the United States. We chose young adults because they use a wider range of social media platforms more frequently than older adults [6], providing us with a deeper understanding of user experience [31]. The participants were aged between 18 and 34 (mean 20.3, SD 3.3) years. Of the 48 participants, 31 (65%) were female. None of the participants chose to drop out from the study, and no cases were excluded from analysis. Of the 48 participants, 13 (27%) were Asian, 5 (10%) were Black, and 30 (63%) were White. They reported using the following social media platforms: Instagram (41/48, 85%), YouTube (31/48, 65%), TikTok (29/48, 60%), Facebook (24/48, 50%), Twitter (subsequently rebranded X; 22/48, 46%), and Reddit (14/48, 29%).

#### Ethical Considerations

This study was determined to be exempt by the institutional review board of the Ohio State University (2020E1164). It was deemed that this study, which involved interview procedures, would not reasonably place the participants at risk of harm. Before the focus group participation, individuals were presented with a consent form, informing them of the purpose of the research. Only those who consented participated in the focus groups. Compensation was given in the form of extra credit at a rate of 0.25 credits per 15 minutes of participation. All analyses involved deidentified data.

#### Procedure

Due to the ongoing COVID-19 pandemic, focus groups were conducted over Zoom (Zoom Video Communications, Inc) using audio and video functions. The focus group topic guide was developed by the researchers, with the overall interview procedure being guided by the work of Krueger and Casey [32]. Each session included 4 to 10 participants and lasted an average of 80 minutes. No parties other than the researchers and participants were present during the sessions. Upon finishing the seventh focus group, the researchers determined that both data saturation and theoretical saturation had been reached; increasing instances of redundancy were found in the data, and successive groups provided diminishing contributions to the development of themes and categories [33]. Thus, data collection ceased after 7 focus group sessions.

The sessions used a semistructured approach, beginning with the identification of unrealistic content. Participants were asked to recall their experiences with, and reactions to, unrealistic content, followed by a detailed discussion on realistic content in later parts of the session. While all 7 focus groups followed this general flow and organization of topics, the specific questions used for each focus group were adjusted to probe the different themes and issues that arose during prior sessions. Given the far-reaching impact of social media on both mental and physical health, the participants were encouraged throughout to consider a variety of content and platforms. At the conclusion of each session, the moderator provided a verbal summary of the discussion, inviting participants to provide feedback on the accuracy of the summary and to offer any additional comments. All sessions were audio-recorded.

#### Data Analysis

Immediately after each focus group, the researchers met to debrief on their notes, considering observed trends, unique concepts, and suggestions for future discussions. Inductive, deductive, and iterative approaches were used. The deductive approach was based on existing research reviewed in the Conceptual Foundation section above [7,8,19,20,23]. Concurrently, because novel themes emerged, coding took an inductive approach: these themes were iteratively reviewed and refined to identify the salient schemas for evaluating social media information and the contexts of this evaluation.

Transcription was carried out manually, with the assistance of Zoom-produced transcripts, and cross-validated with the audio recordings. The transcripts for the 7 sessions comprised 76,704 words in total. Prominent themes across the focus group sessions were identified using the constant comparison approach [34]. NVivo software (Lumivero) was used to code responses by speaker (ie, as a form of case) and to attach participant information such as race, sex and focus group session number as case attributes. The data were chunked into smaller units, and new patterns were compared across successive focus groups using an emergent-systematic design. Specific quotes were coded to each theme in NVivo, allowing for the easy consolidation and creation of the schemas of interest.

As all authors (faculty and graduate students) study the effects of media on health outcomes, we used our knowledge about research findings in this area to inform the interpretation. All researchers, comprising female and male individuals, were involved in the hosting of the focus groups and the theme development process. The COREQ (Consolidated Criteria for Reporting Qualitative Research) checklist is provided in Multimedia Appendix 1.

### Results

#### Overview

Overall, the focus group research revealed how social media users judge the realism of content. Multiple approaches were identified: falsity, authenticity, naturality, resonance, and social assurance. Textbox 1 presents conceptual definitions of these schemas.

**Schemas for evaluating social media content.**

**Schema and definition**

- Falsity: general sense of distrust or doubt about social media content
- Naturality: willingness to accept artificiality and engineered aspects of social media representations
- Authenticity: the extent to which one’s social media posts align with one’s inner thoughts and feelings
- Resonance: felt relatedness between a social media post and the user’s self
- Social assurance: reliance on metrics associated with social media posts

#### Falsity

There was a general sentiment that much of what is on social media is not real, or at least some of it cannot be trusted. A pervasive sense of disillusion and doubt was evident throughout the focus group sessions. Many participants assumed that most, if not all, of what is on social media is not real or at least does not present “the whole picture.” This ranged from fake news (usually fake unless coming from a credible platform with citations and verification) to accounts with few followers, “weird” usernames, low-quality pictures in profiles and posts, no mutual followers, and repetitive posts. The motivation for these behaviors was considered to be for money, fame, popularity, and a need for attention. These specific visible cues and concrete motivations associated with the widespread falsity of information on social media were noted by participants consistently across the 7 focus group sessions.

In addition to these more surface-level determinants, participants’ comments revealed another layer. They perceived that there may be a “fake ideology” on social media that can easily create a “false perception” of somebody:

Everyone just has this invisible hand pushing them to do something and not do something.

P8; male; group 2

There’s a lot of like performative activism. I’m not sure if that’s the right word, but people just post things because they want to make themselves look better, not because they actually believe in whatever.... So, it’s like really hard to tell sometimes on social media versus real life.

P10; female; group 2

Regarding a more subtle level of fakeness, participants showed a certain level of resignation, tolerance, and acceptance of this aspect. They said that they tend to ignore it or to not pay attention:

I try not to read too much into other people’s posts.... I know I put a disclaimer on my Facebook that says that I’m showing more mountain tops than the valleys that I also have in my life, like everyone else.

P14; female; group 2

Others were concerned that the lack of realism on social media can have “toxic” effects:

I think that it is generally unimportant for people to be realistic on social media, and it has given us a false perception of the way people are in our communities, in our society.

P8; male; group 2

#### Authenticity

Participants expressed concerns about the degree to which the content posted by others is a true representation of the self. They cared about the extent to which an individual’s posts reflected their internal thoughts and values. Content that demonstrated authenticity was perceived as more real. Whereas 1 facet of falsity involved conforming to social norms and “invisible hands,” authenticity was seen as asserting one’s uniqueness:

Posts that I think are genuine [are ones] about trying to not be like everybody else.

P41; female; group 6

Personally, I like real content like...if you’re being authentic then you, I think you can build like a friendship or bond with that person because you’re being vulnerable with each other. Versus like if you’re just fake all the time like you’re posting Photoshopped images like you’re in it to look good. You’re not, you’re in it for the popularity points I think instead of the friendship or the realness.

P38; female; group 6

Participants provided examples of authentic content. Self-disclosure was widely regarded as a signifier of real content. It did not need to focus on negative experiences, although some found that events “like arguments and stuff like that...are more likely to be real than people being nice to each other... [since they are] harder to fake” (P29; female; group 5). Another participant said that realism was conveyed through the disclosure of one’s “deep emotions or secret struggles...or personal issues” because “[it] makes you feel closer to that person, or like you know them a little bit better as a person” (P31; female; group 5), and yet another participant added that “it’s like really getting more insight of them personally instead of [just seeing] their outside positive side” (P34; female; group 5). Disclosure provided a bridge between an individual’s inner thoughts and outer expressions, allowing them to be consistent to their true self. This evaluation applied to public figures as well:

People always put their best foot forward on social media, usually.... So, I think I get an indicator that something is real when an influencer takes a minute and says, “Here’s a life update: I actually haven’t been the happiest.” I tend to believe that those things are real, just because people don’t often open up like that on social media.

P31; female; group 5

#### Naturality

Although participants generally wanted others’ presentation of the self and events to mimic external reality and its flaws, they made allowance for some degree of latitude and deviation. Participants indicated a certain willingness to accept some of the artificial and engineered aspects of self-presentation and storytelling that digital media affordances allow.

At a basic level, the practice of editing visual content such as photos or videos had implications for perceptions of naturality. Participants seemed to embrace a tolerable level of artificiality, as they distinguished between “heavy,” “extreme,” and “body-altering” editing from “enhancements.” The former were generally regarded as unreal, treated with disapproval or even light ridicule. Multiple participants also indicated an ease with identifying these forms of heavy editing, that “it’s really obvious” (P27; male; group 4). By contrast, administering enhancements such as color corrections and “basic” filters was regarded as not only acceptable but also a predominant practice on the web. Content was still considered naturally real as long as edits were unobtrusive and refrained from altering the substance of the photos, such as erasing portions of the background or morphing body parts.

As a participant described, these types of edits are meant “to change the quality [of the image] instead of changing the actual person” [in the post].... Everyone does that” (P21; female; group 4). Filters serve to “make [photos] pop out and bring [them] to life a little more,” allowing audiences to “perceive what [the posters are] seeing, what [they are] trying to display” (P5; male; group 1). In other words, rather than strictly adhering to factual realism by refraining from manipulating the content, users might instead provide naturality through enhancing edits. These changes intentionally overemphasize features of the post to compensate for the experiential gap between the poster and viewer. Thus, enhancing edits demonstrate naturality by maintaining a balance between fidelity to reality and (unobtrusive) manipulation.

#### Resonance

A sense of resonance was another lens through which participants evaluated realism. The degree to which the social media representation reverberated with one’s own experience was important:

I feel like we base what’s real or not...on what our own experiences are. So, if we’ve experienced something similar...through ourselves or other people, we’re more likely to see it [as real]. So, if no one that we know closely has ever done X, Y, or Z, then we’re less likely to believe that another person outside of our circle has ever done that.

P29; female; group 5

Another participant explained that relatable content felt more real:

[L]ike when people like sharing more personal events on their Instagram or YouTube, I feel like people are able to relate to it more, and I think it makes it more believable, the message they’re trying to put out.

P1; male; group 1

In this context, participants tended to perceive content from peers as more realistic because these sources were considered more relatable. Peers were seen as relatable because they shared similar traits and life experiences with the participants; for instance, a participant described how he tended to “compare [himself] way more to people [his] own age and people in college than [to] celebrities” (P27; male; group 4). In this case, the participant’s identity as a young adult and a student served as a standard for comparison to other social media users.

The benefit and value of resonance were described by another participant:

So, when people see others talking about what’s going on in their lives or something that they relate to, and seeing in a context that it’s okay to feel this way. It’s okay to be this way. It’s okay to look this way. It’s okay to be like, who you are in this facet. The more exposure they have to that type of like positivity and acceptance, the more widespread it is, so, I feel like realness helps others come to terms with their own selves.

P20; female; group 4

#### Social Assurance

Another factor in determining the realism of social media content was the degree of social validation associated with it. Online activity data, which serve as a signifier that others have endorsed the content, influenced the evaluation of social media content. The visibility of others’ engagement behaviors may indicate an existing consensus regarding the realism of the content. Preexisting engagement with a post can influence users’ perceptions of the content’s validity by suggesting how real others have already determined it to be.

Participants reported that seeing the interactions of others influenced their perceptions of realism; for instance, a participant described how engagement metrics caused her to reconsider her initial evaluation of a post because after seeing “all of [her close friends and family] posting positive comments and stuff like that, it [made her] think... [she] should like this post [too] because they believe it” (P10; female; group 2). This participant also noted how a *lack* of likes or comments on a post sowed discomfort and doubt about the realism of the post;

If I do see a post that I really like, and nobody has commented on it yet, I feel weird. I don’t want to be the first one [to interact with it].

P10; female; group 2

Engagement metrics such as likes, comments, and shares influenced users’ assessments and advocacy, or lack thereof, of the realism of certain content, biasing subsequent evaluations from other users.

### Discussion

#### Overview

The 5 main themes that emerged from the focus group sessions suggest that users manage divergent schemas to assess the relationship between social media and reality. The binary distinction of fake versus fact may not appropriately capture these nuances. While participants expressed that they were distrustful of social media content, this schema coexisted with others, which allowed for deviations, different standards, and less rigorous applications. Altogether, these schemas suggest that users are willing to negotiate the boundaries between social media and reality based on the context and motivations.

Of note, these findings build upon and extend previous research on several key areas, including the effects of mass media on health behaviors [7,14,15] and authenticity [18-20] or social media metrics [22-24] pertaining to health-relevant decisions and actions. The results illuminate the distinct nature of judgments regarding social media realism compared to mass media realism. The findings underscore that the evaluation of social media realism encompasses dimensions that go beyond the consideration of authenticity or social media metrics. This study advances our understanding of how individuals perceive digital information, suggesting multiple pathways through which social media information can impact health behaviors.

#### Dimensions

Falsity may reflect an overarching skepticism toward social media content. This concern ranged from the more visible factors, including fake accounts using weird usernames without a profile photo, to the more subtle issues, including people not being honest on social media. This latter concern was expressed using terms such as “fake ideology” and “invisible hands” on social media that drive people to be less than forthright.

The remaining 4 dimensions could be grouped into 2 categories. The first category includes authenticity and naturality, which concern the closeness between true others and their social media representations. Authenticity may be more about the inner thoughts and feelings of others, whereas naturality focuses on their external features and characteristics. The second category includes social themes, including resonance and social assurance. Resonance is based on a deeper level of understanding of others and the self, whereas social assurance relies on surface-level cues created by others.

Naturality refers to the tolerable degree of departure from the true forms and shapes of entities in reality. It is a willingness to accept a certain level of artificiality, straddling a line between the original and genuine and the made-up, manufactured forms and shapes in our world. These findings echo prior research reporting that “no-makeup” movements and calls for “natural beauty” are actually positively associated with cosmetic sales [35]. Users seem to place a premium on the appearance of naturality, even when it is a product of planned and contrived efforts. This openness to compromise seems to be stronger when social media use is motivated by enjoyment rather than surveillance. Some participants even noted that realism does not matter on these occasions; for example, although some participants described TikTok as fake, others defended it as a platform where they can be silly and have a good laugh.

Authenticity refers to the extent to which a person’s social media representations align with their true self. It entails evaluating the consistency between the 2 [15]. Authenticity requires the willingness to be genuine and distinguish oneself from others, while falsity often arises from conforming to what is trendy, popular, and acceptable on social media. Users expect authentic self-representations to be consistent and stable over time and not be influenced by the social norms of superficiality and positivity.

Resonance is the self-validation that one derives from others’ content and posts. It occurs when others’ posts reverberate with one’s own experiences, beliefs, and attitudes. Whereas authenticity refers to the alignment between the true and social media–constructed self, resonance is the similarity of self and others on social media. Users find the feelings of connection and relatability meaningful and rewarding because they provide opportunities for reflection and validation of the self and their thoughts and experiences. Therefore, resonance refers to the relatedness between a post and the user’s self that is felt internally.

By contrast, social assurance is a heuristic that users use to quickly judge the veracity and social value of a message. Unlike the assessment of authenticity and resonance, which may require a relatively closer examination of social media content, social assurance relies on more readily visible metrics associated with a post, including likes. Knowledge of other users’ engagement with a post may serve as cues for the credibility [23] as well as general acceptance of the content [22]. As a contrast to mass media, these findings highlight the value of shared experiences on social media.

In summary, the widespread concern about falsity coexists with the willingness to accept manufactured social media representations by others and rely on others’ judgments rather than one’s own. While users may be suspicious about others’ posts and motives, they still value authenticity and report benefiting from resonance experiences. Moreover, the focus group discussion suggests that these schemas may be related to social media use motivations, gratifications, and the contexts that different platforms differentially afford.

#### Limitations

There are some limitations to this study. The participants were college students, who may not represent the broader population of social media users. This sample was chosen because young adults use social media more frequently and on a wider range of platforms than older adults. As samples of experienced users allow researchers to gain a more in-depth insight into user perspectives [25], these participants may appropriately capture the purpose of this focus group research. Future research should include a more heterogeneous sample. Due to the focus of this paper, we could not include all contextual information that emerged during the focus group sessions. Finally, focus group data involve the communication of ideas within a social setting. Participants may express their thoughts differently within a social setting compared to a private or anonymous setting.

## Study 2: Quantitative Survey

### Overview

The focus group research provided valuable insights into the diverse approaches that people use to navigate the array of information on social media. On the basis of these findings, we developed measurement items capturing users’ schemas for evaluating social media content. The items were then administered to a general sample of social media users to initially assess their content validity and construct validity.

### Hypotheses

#### Overview

The results of our focus group research showed that social media reality judgments may involve 5 dimensions: falsity, naturality, authenticity, resonance, and social assurance. On this basis, we formulated the following hypothesis:

- H1—Perceived social media realism is a multidimensional construct comprising falsity, naturality, authenticity, resonance, and social assurance.

To test the distinctiveness of the dimensions, we formulated hypotheses predicting the relationship between each dimension and ≥1 external variables. Informed by the insights gained from our focus groups and literature review, we expected that the schemas would be differentially associated with features of social media use.

#### Falsity

We considered that falsity would be distinct from the other dimensions in that it is a general sense of distrust about what is presented on social media. Despite this skepticism, the participants in our study, as well as those in a global study on information literacy [4], continued to use social media. However, the frequency and level of engagement may differ, depending on the strength of the falsity beliefs. Some participants noted that they do not “read too much into” others’ posts, while other participants reported closing their accounts on certain platforms. For these reasons, we hypothesized that falsity will be negatively associated with the frequency of use and engagement with social media. We also expect that stronger falsity beliefs would lead to greater information seeking to verify the social media representations against established facts. Information seeking is a behavior to reduce uncertainty [36], which could be associated with suspicion about the veracity of the stimuli at hand. Accordingly, we formulated the following hypotheses:

- H2a—Falsity will be negatively associated with use frequency and engagement with social media.
- H2b—Falsity will be positively associated with greater information seeking.

#### Authenticity

We thought that the anticipated multiple dimensions should be differentially associated with the currently available global measure of perceived social media realism [18,19]. This existing global measure, as discussed earlier, seems to primarily capture authenticity, which is one of the most frequently studied aspects of social media realism judgments. Therefore, we anticipated that the extant global measure will be more strongly associated with authenticity than with the other dimensions. We anticipated that the beliefs about authenticity could be linked to eudaimonic gratification from social media use. Research has found that one’s psychological well-being is associated with the sense that one can manage and maintain one’s authentic self [37]. Building on these findings, we conjectured that the desire for, and rewards from, encountering and interacting with authentic others could lead to a meaningful social media use experience, thereby facilitating eudaimonic gratification. Accordingly, we developed the following hypotheses:

- H3a—Authenticity will be more strongly associated with the existing global measure of realism than the other dimensions.
- H3b—Authenticity will be positively associated with eudaimonic gratification.

#### Naturality

Naturality, the willingness to embrace artificiality in social media representations, may be related to other types of gratifications or motivations. We hypothesized that the reliance on the naturality schema may be associated with hedonic enjoyment gratification and the motivation to escape from the mundane and routine world through social media use. During our focus group sessions, some participants commented that realism does not really matter when they want to have fun being on social media. This pursuit of happiness through media use has been conceptualized as hedonic enjoyment [38]. Escape is the desire to get away from the daily rituals of work and life [39]. In this state, people may prefer new, novel, and exotic stimuli to the familiar [40]. Accordingly, the following hypotheses were constructed:

- H4a—Naturality will be positively associated with hedonic gratification.
- H4b—Naturality will be positively associated with escape motivation.

#### Resonance

As users manage networks that influence the kinds of information they will consume, network characteristics may be related to the schema they use to evaluate social media messages. The preference for ideas that affirm and reinforce existing ones—resonance—may be associated with a homophilous social media network. Homophily refers to the degree of similarity of node attributes, reflecting the socialization process and social attitudes [41]. The propensity to value consonant social media information may be linked to how individuals use the information [27]. Specifically, confirmation bias was found to be positively associated with the time spent processing information and the degree of cognitive reflection on the information [28]. These findings suggest that resonance may predict greater reflective thinking when exposed to self-affirming information. Accordingly, we formulated the following hypotheses:

- H5a—Resonance will be positively associated with homophilous social networks.
- H5b—Resonance will be positively associated with greater reflective thinking.

#### Social Assurance

Social assurance, the dependence on external metrics rather than the internal message content to form judgments, may serve a surveillance goal when using social media, that is, when a person’s primary purpose is to find out what is going on in society and what is popular and trendy, external metrics can be useful. However, this dependency on mental shortcuts of social assurance is unlikely to be positively associated with either information-seeking or reflective-thinking behaviors. The following hypotheses were developed:

- H6a—Social assurance will be positively associated with surveillance motivation.
- H6b—Social assurance will not be associated with information seeking or reflective thinking.

#### Research Question

In addition, we wondered whether these 5 schemas of realism judgments might be differentially related to the addictive use of social media [42,43]. Prior research found that perceived social media realism is a factor predicting social media addiction [44]. This finding suggests that correcting social media realism may mitigate social media addiction. However, the study used a global measure of realism, and it is unknown which specific dimension may have a stronger association. As little prior research provided us a basis to formulate a hypothesis, we proposed the following research question (RQ) to examine the possible associations:

- What are the relationships between the 5 dimensions and social media addiction?

### Methods

#### Design and Participants

We recruited social media–using adults through the CloudResearch web-based panel (Prime Research Solutions LLC). To ensure a diverse sample, we used quota sampling to ensure that our sample comprised 50% young adults (aged 18-29 y) and 50% adults (aged ≥30 y). This decision was based on research indicating that younger adults are more likely to use social media than older adults [6]. The sample size was 442 participants, after excluding those who failed 1 of 4 attention checks (n=28). The participants’ ages ranged from 18 to 96 (mean 41.8, SD 20.7; median 29.0) years. Of the 442 participants, 219 (49.5%) were female. They reported using the following social media platforms: Facebook (392/442, 88.7%), Instagram (290/442, 65.6%), Reddit (180/442, 40.7%), TikTok (234/442, 52.9%), and Twitter (213/442, 48.2%).

#### Ethical Considerations

This study was determined to be exempt by the institutional review board of the Ohio State University (2022E0763). It was deemed that this study, which involved survey procedures, would not reasonably place the participants at risk of harm. Before the survey participation, individuals were presented with a consent form. Only those who consented participated in the survey. The web-based panel company CloudResearch provided the compensation to participants, which was up to US $3. All analyses involved deidentified data.

#### Measures

Measures were given on a scale ranging from 1=*strongly disagree* to 5=*strongly agree*, unless noted otherwise. On the basis of the findings from the focus groups and existing research, items were developed to measure the 5 aspects of social media reality judgments: falsity, naturality, authenticity, resonance, and social assurance. Multimedia Appendix 2 presents the full list of items, along with scale means, SDs, and reliability.

Social media use frequency was assessed for 5 platforms, including Facebook and Twitter. For each platform, participants were asked how often they used it, and they rated their use on a 5-point scale: 1=*I don’t use this*, 2=*seldom*, 3=*sometimes*, 4=*often*, and 5=*very often* (mean 2.94, SD 1.09; Cronbach α=0.72). Social media engagement was measured by asking participants how often they engaged in liking, commenting, replying to others’ comments, and sharing. The scale ranged from 1=*never* to 5=*very often* (mean 3.44, SD 0.91; Cronbach α=0.84).

With regard to information seeking, following the stem “after seeing others’ social media posts,” participants were given items that included how often they “look up additional information to know more about the topic.” The scale ranged from 1=*never* to 4=*often* (mean 2.77, SD 0.74; Cronbach α=0.85).

Items for the global measure of social media realism included “What’s on social media reflects real life for the most part” (mean 3.01, SD 0.95; Cronbach α=0.90) [18].

Gratifications included eudaimonic and hedonic enjoyment. Items were adapted from the study by Oliver and Raney [38]. Eudaimonic gratification items included social media “provide a greater understanding of life” (mean 3.41, SD 0.89; Cronbach α=0.86). Hedonic enjoyment items included “I have fun while on social media” (mean 3.79, SD 0.78; Cronbach α=0.85).

Motivations included escape and surveillance. Escape motivation was measured with the scale developed by Rubin and Perse [39]. Following the stem “I use social media to,” items included “forget about work and other things” (mean 3.41, SD 0.99; Cronbach α=0.90). Surveillance motivation was assessed using the social media use motivation scale developed and validated by Cho et al [19]. Respondents indicated the degree to which they use social media to “keep up to date with what is trending” (mean 3.48, SD 0.87; Cronbach α=0.84).

Network homophily [45] was assessed by presenting the stem “most people in my social media connections” and following it with items that included “have thoughts that are similar to mine” and “express attitudes similar to mine” (mean 3.49, SD 0.80; Cronbach α=0.83).

Regarding reflective thinking, participants were asked, “When seeing other people’s social media posts, how often do you think about the following?” Items included “what may be the motivation for the post,” and the scale ranged from 1=*never* to 4=*often* (mean 2.82, SD 0.67; Cronbach α=0.84). These items were based on the study by Chen [46].

Addictive social media use was measured with 6 items taken from the study by Caplan [42]. The items represented compulsive use, problematic use, and withdrawal dimensions. As these items loaded onto the same factor (loadings: 0.75-0.85), we combined them into a single factor (mean 2.53, SD 0.99; Cronbach α=0.88).

#### Data Analysis

H1 was tested with confirmatory factor analyses (CFA) using the R package *lavaan*. H2 to H6 and the RQ were tested using a series of ordinary least squares multiple regressions. In each model, the dependent variable was regressed onto all dimensions, rather than only the one predicted to be associated, for a more rigorous evaluation. No issue of multicollinearity was found in the data, with variance inflation factors <4, tolerance values >0.2, and condition indices <30. All analyses were conducted using R (R Foundation for Statistical Computing).

### Results

#### Factor Structure

H1 hypothesized that the items would comprise a 5-factor structure. Participants’ responses to the items were included for the CFAs. First, a first-order single-factor model was estimated. Second, a first-order oblique 5-factor model was estimated, where the 5 factors were allowed to be correlated with each other. Third, we estimated a first-order 3-factor model comprising (1) falsity, (2) naturality and authenticity, and (3) resonance and social assurance. Finally, a second-order single-factor model was estimated, where the second-order factor loaded on the 5 first-order factors, which were not allowed to be correlated.

**Figure 1:**
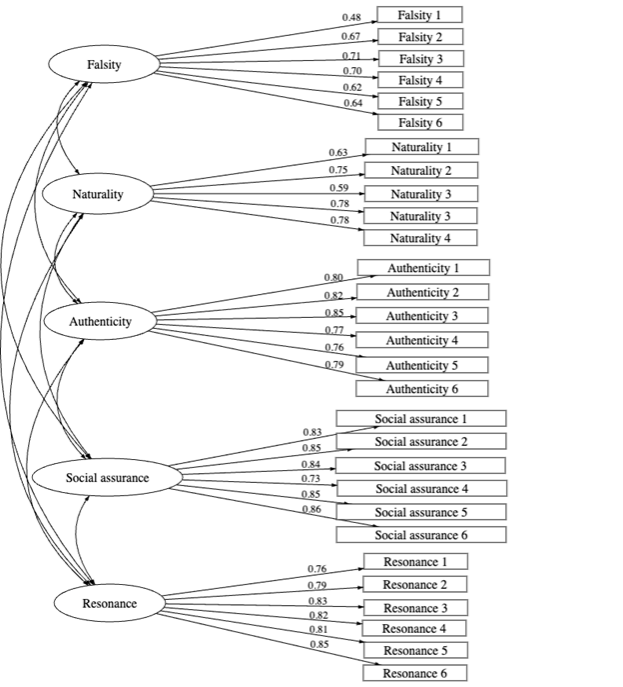
Factor structure.

Three indices evaluated the fit of the CFA models: the comparative fit index (desirably >0.90) [47], the root mean square error of approximation (desirably <0.08) [48], and the difference of Bayesian information criterion across the models [49]. Model comparison was performed using sequential likelihood ratio tests. Table 1 shows the model fit indices. These results show that the first-order oblique 5-factor model yielded the best fit to the data. The model comparison demonstrated that the first-order 5-factor model performed significantly better than the first-order single-factor model (Δ*χ*^2^_10_ =2556.1; *P*<.001), the first-order 3-factor model (Δ*χ*^2^_7_ =1326.6; *P*<.001), and the second-order single-factor model (Δ*χ*^2^_5_ =51.0; *P*<.001). The best of these 4 models is therefore the first-order 5-factor model. This model also has the lowest information criteria of all models (Akaike information criterion=27,890, Bayesian information criterion=28,168), which suggests that it is the best fitting model out of the 4. These findings support H1. Table 2 summarizes the results, where the numbers are standardized regression coefficients. Figure 1 presents the factor structure.

**Table 1:** Confirmatory factor analysis results.

Model	Chi-square (*df*)	CFI^a^	RMSEA^b^
First-order single-factor	3519.9 (377)	0.606	0.137
First-order 3-factor	2290.4 (374)	0.760	0.108
First-order 5-factor	963.8 (367)	0.925	0.061
Second-order single-factor	1014.8 (372)	0.919	0.063

^a^CFI: comparative fit index.

^b^RMSEA: root mean square error of approximation.

**Table 2:** Ordinary least squares regression results of testing H2 to H6 and the research question.

	Falsity	Naturality	Authenticity	Resonance	Social assurance
SM^a^ use frequency	0.199^b^	0.257^b^	0.065	0.095	0.230^b^
SM engage	0.128^c^	0.213^b^	0.163^c^	0.208^b^	0.064
Info seek	0.197^b^	0.138^c^	0.082	0.248^b^	0.054
Global	0.004	0.012	0.467^b^	0.142^b^	0.267^b^
Eudaimonic	0.114^c^	0.138^c^	0.175^b^	0.303^b^	0.085
Hedonic	0.023	0.321^b^	0.188^b^	0.312^b^	–0.091
Escape	0.219^b^	0.228^b^	–0.024	0.119^d^	0.280^b^
Homophily	0.102^d^	0.047	0.242^b^	0.201^b^	0.150^d^
Reflect think	0.105^d^	–0.009	0.135^d^	0.266^b^	0.107
Surveillance	0.049	0.155^b^	0.076	0.277^b^	0.193^b^
Addictive use	0.062	0.102^d^	0.101^d^	0.041	0.469^b^

^a^SM: social media.

^b^*P*≤.001.

^c^*P*<.01.

^d^*P*<.05.

#### Relationships With External Variables

Zero-order correlations between the variables are provided in Multimedia Appendix 3. Table 2 presents the results of testing H2 to H6 and the RQ.

H2a predicted that falsity would be negatively associated with social media use (frequency and engagement), and H2b predicted that falsity would be positively associated with information seeking. H2a was rejected because falsity was positively linked to social media use frequency and engagement. On the other hand, falsity positively predicted information seeking. Other dimensions, including resonance, predicted information seeking. H2b was partially supported.

H3a expected that authenticity would be positively associated with the existing global measure of social media realism, and H3b expected that authenticity would be positively associated with eudaimonic enjoyment. Supporting H3a, authenticity was substantially related to the global realism measure, with other dimensions showing weaker associations. Authenticity positively predicted eudaimonic enjoyment, but so did resonance. These results provide partial support for H3b.

H4a hypothesized that naturality would be positively associated with hedonic enjoyment, and H4b hypothesized that naturality would be positively associated with escape motivation. Naturality was indeed positively associated with hedonic enjoyment and escape motivation. Hedonic enjoyment was also predicted by other dimensions such as resonance. Escape motivation was related to other dimensions, including social assurance and falsity. H4a and H4b were partially supported.

H5a anticipated that resonance would be positively associated with social media network homophily, and H5b anticipated that resonance would be positively associated with reflective thinking. Resonance was positively associated with social network homophily. However, other dimensions, including authenticity and social assurance, were also linked to network homophily. H5a was partially supported. Supporting H5b, resonance showed a stronger positive association with reflective thinking than any other dimensions.

H6a hypothesized that social assurance would be positively associated with surveillance gratification, and H6b hypothesized that social assurance would not be associated with information seeking or reflective thinking. Social assurance was positively associated with surveillance gratification; other dimensions, including resonance, were also linked to surveillance. These findings partially support H6a. Consistent with H6b, social assurance showed no association with information seeking or reflective thinking.

The RQ asked whether the 5 dimensions would be differentially associated with addictive social media use. Social assurance was more strongly associated with addictive social media use than the other dimensions. Authenticity also showed a positive association. Falsity, naturality, and resonance were unrelated to addictive social media use.

### Discussion

#### Summary

This survey study provides initial quantitative evidence that social media reality judgments comprise 5 dimensions—falsity, naturality, authenticity, resonance, and social assurance—and that these dimensions are differentially associated with the patterns of social media use.

#### Dimensions

Falsity was the strongest belief among the dimensions, as shown in Table 2. The strength of this schema observed in this study may reflect the finding of a recent global study on information literacy [4] that the majority of social media users are aware of the problem of misinformation. However, this belief did not deter people from using, and engaging with, social media. An alternative explanation may be that frequent social media users are more likely to encounter misinformation. Future research should examine this potential using a longitudinal design. By contrast, falsity did motivate users to seek information and engage in reflective thinking. The falsity schema may function as a general self-protective mechanism against potential harm on social media. The fact that falsity was related to escape motivation suggests that less purposeful and more passive use of social media [50] may present users with less-than-credible information.

Naturality is a unique dimension that emerged in this study, representing openness to artificiality in social media presentations. This schema was linked to hedonic enjoyment and escape motivation for social media use. Naturality may be particularly relevant when people use social media for entertainment and diversion purposes because they may be more willing to embrace the use of filters, digital editing and alterations, and preplanned and prechoreographed videos. As hedonic gratifications are central to social media use [51], it is important for future research to understand the mechanisms and effects of naturality. Furthermore, the schema of naturality can be useful for research on visual misinformation and visual information processing, with concerns growing over the increasing prevalence of deep fakes and cheap fakes [52].

Authenticity, as hypothesized, was more strongly associated with the current global measure of social media realism than the other dimensions. This finding highlights the necessity for multidimensional measures, such as the ones proposed in this study, for a more comprehensive understanding of user judgments of social media realism. Concurrently, the results show that authenticity plays important and multiple roles in social media use. It was positively linked to eudaimonic and hedonic enjoyment, but the belief that people’s social media posts represent their true selves also predicted addictive social media use.

Resonance is another distinctive dimension identified in this study. It was a stronger predictor than the other dimensions of reflective thinking and information seeking after social media use. This finding corroborates the confirmation bias theory, which argues that people tend to search for, and focus on, information that supports their preexisting beliefs. Perhaps as a result, resonance facilitated social media engagement, but it was unrelated to the frequency of use. In light of these findings, future research should investigate how users’ social media networks influence resonance. Network homophily was associated with resonance in this study. Future research should expand on this result to identify other network features.

Social assurance, a heuristic-based judgment, was unrelated to reflective thinking or information seeking. It was positively associated with surveillance motivation for keeping up with what is trendy. Social assurance was a stronger predictor than the other dimensions of addictive social media use, suggesting that it may have a less-than-healthy role in social media use. While unrelated to eudaimonic or hedonic enjoyment, social assurance was positively associated with escape motivation. This schema was positively linked to the frequency of social media use but not to engagement with social media. These results suggest that people depend on social assurance when they engage in passive use of social media.

The results offer further perspectives on the distinctiveness of the dimensions, as well as their connectedness. Although resonance and social assurance share a social-orientation focus, the results show that they function starkly differently. Resonance promoted reflective thinking and information seeking, while social assurance was unrelated to either. The former fostered eudaimonic and hedonic enjoyment, but the latter did not. Social assurance was strongly linked to addictive use, but resonance was not. Resonance predicted engagement, while social assurance was related to the frequency of use. Resonance is an internally sensed connection between a post and the user, whereas social assurance is a dependence on external cues.

Authenticity and naturality are both judgments about others, but the former focuses on internal thoughts and feelings, while the latter focuses on external features, of others. Authenticity was strongly associated with the existing global measure of social media realism, while naturality was not. Considering the important role of naturality evidenced in this study, more research is needed to examine this dimension and its effects on digital media uses and outcomes.

In addition, our findings suggest that resonance can be an important dimension influencing the effects of social media use on health-related outcomes. Beyond our predictions, resonance was linked to the gratifications and motivations related to eudaimonic well-being, hedonic pleasure, and surveillance. These associations demonstrate the importance of the sense of connection with others in social media use and its impact on how individuals evaluate digital information. Consistent with our expectation, resonance predicted reflective thinking more strongly than the other dimensions. This finding suggests the central role of resonance in influencing how individuals may engage with health information on social media. Collectively, these findings highlight the need for future research to closely examine the role of resonance in determining the evaluation of health information on social media. Resonance is likely based on one’s prior experiences and beliefs, which can bias subsequent judgments and actions [53].

#### Limitations

This study has limitations. As it used a cross-sectional design, making a causal inference is difficult. Although the predictions were based on theory, findings should be interpreted keeping this limitation in mind. Future research could apply the measures to diverse platforms and to more specialized populations to examine how these differences may shape the schemas. The affordances of a given platform may promote the use of certain schemas rather than others. There may be differences in schema use across social media use experiences.

The results of this survey study provide a better understanding of social media reality judgments. The results further illustrate that the different schemas may coexist in users for different goals, gratifications, contexts, and outcomes. This understanding could be useful for future efforts for digital media literacy education to prevent the harmful effects of various forms of misinformation, including visual misinformation.

## General Discussion

### Principal Findings

Across the 2 studies, this research identified prominent user schemas for evaluating social media reality and investigated the schemas’ association with social media use behaviors. These efforts aimed to develop and preliminarily validate a social media reality measure, an instrument designed to capture multifaceted user schemas used in assessing social media messages. Given the pervasiveness of misinformation, our goal was to provide a general measure that can be used in diverse domains of public health. The areas of application can include, but are not limited to, a wide range of public health issues such as drugs and smoking, communicable and noncommunicable diseases, diet and eating disorders, treatments and medical interventions, and mental health [2,3,7,9-11,14-19,21,24,42-44].

The development of these measures opens up possibilities for future research on social media and public health. The multifaceted user schemas suggest a new avenue for enhancing current efforts to counter misinformation through tailored digital media literacy initiatives. The 5 dimensions suggest that research may need to move beyond the current binary distinction of fake versus fact to obtain a more dynamic understanding of the user interpretations of social media information. On the basis of this understanding, user-centric tailored digital media literacy interventions can be developed.

Through formative research, interventions can specifically identify whether users have low or high levels of these schemas; for example, for users with high levels of beliefs on certain dimensions (eg, naturality, resonance, and social assurance), tailored interventions can seek to reduce the beliefs by providing facts, statistics, or anecdotal evidence. Conversely, for users with low levels of beliefs on dimensions such as falsity, a tailored intervention can focus on content that provides new information to increase these beliefs. For users with the desirable levels of beliefs (eg, low levels of beliefs on social assurance), interventions can focus on maintaining or reinforcing the beliefs. The optimal level of beliefs for each dimension will depend on the specific public health issue and context, necessitating a user-centric approach. The scales developed in this study can usefully assist these efforts.

In this approach, educators can also encourage users to critically examine and discern the various facets of a social media post and understand how the facets impact their judgments and actions; for instance, a funny TikTok video may be deemed strongly authentic, although not highly natural. A glamorous Instagram image may be perceived as natural yet lacking in authenticity. By challenging users to more critically examine their beliefs, educators can help users recognize how their existing beliefs and values shape their evaluations and improve their ability to differentiate social media information from reality.

By using these scales, researchers can also examine how users deal with diverse messages within the contexts of differential motivations and gratifications. The findings of this study (eg, users being more willing to embrace artificiality in social media presentations when motivated by escapism) can be used in educational efforts so that users can better understand how their evaluation is context dependent.

The findings suggest that the multiple functions served by each schema in various contexts and goals should be acknowledged. The results and the social media reality measures of this study can assist educators in assessing users’ existing schemas and the context within which the schemas operate; for example, although perceived authenticity may provide enjoyment, it was weakly linked to the addictive use of social media. Therefore, users can be prompted to question their assumptions about authenticity. Likewise, users who hold beliefs about naturality may be obtaining hedonic enjoyment, but they may also be vulnerable to the growing presence of visual misinformation on social media, including deep fakes and cheap fakes.

The diametrically different functions of the 2 socially focused schemas should also be addressed. The use of the resonance schema can be rewarding, but its stronger association with reflective thinking and information seeking compared to falsity suggests that it may reinforce confirmation bias. Digital media literacy efforts can inform users of this tendency and encourage openness to different opinions. Users who rely on social assurance should critically examine the content they quickly accept based on metrics generated by others.

In addition, research could use the scales to examine the role of the schemas in the mechanisms of social media effects because different schemas, when primed, may activate different types of information processing and produce differential outcomes.

Although the initial form of the social media reality measure would benefit from further validation, it holds potential for future applications. To improve on our research, future research could use more diverse samples and use a longitudinal design that allows causal inference of the realism effects.

The results of this research advance our existing knowledge. They demonstrate that judgments of social media realism differ significantly from those of mass media realism [7,8]. The findings also indicate that the dimensions are broader than the current focus on authenticity [18-20] or social media metrics [23], encompassing aspects such as falsity, naturality, and resonance. Furthermore, the results illuminate how these different dimensions are differentially connected to health-relevant perceptions and practices, including information seeking and reflective thinking, social media network structures, and social media addiction. These insights suggest avenues for modification and correction. By unpacking the complexity of social media content evaluation, research can better develop tailored interventions and strategies that improve digital media literacy and foster healthier web-based engagement.

### Conclusions

The schemas identified in this research contribute to a more nuanced understanding of how users interact with, and interpret, social media. We hope that these studies will stimulate further research toward a deeper and more dynamic understanding of user evaluations of social media information. Understanding where users stand regarding these schemas can be a basis for developing personalized digital media literacy education tailored to each individual’s distinctive set of schemas and for proactively shielding users from the harmful influence of misinformation on social media.

## Appendix

Multimedia Appendix 1 COREQ (Consolidated Criteria for Reporting Qualitative Research) checklist.

Multimedia Appendix 2 Dimensions and items.

Multimedia Appendix 3 Bivariate correlations between variables.

## Abbreviations

CFA: confirmatory factor analyses
COREQ: Consolidated Criteria for Reporting Qualitative Research
RQ: research question
