# Supplementary material for: A Multidimensional Approach for Evaluating Reality in Social Media: Mixed Methods Study
Source: J Med Internet Res. 2024 Aug 6;26:e52058. doi: 10.2196/52058 (PMC11336507; doi:10.2196/52058)
Supplement: Multimedia Appendix 2 [file jmir_v26i1e52058_app2.docx]

**Multimedia Appendix 2**

**Dimensions and items**

| Dimensions | Items | M | SD | α |
| --- | --- | --- | --- | --- |
| Falsity |  | 4.11 | .53 | .80 |
|  | Misinformation is everywhere on social media. |  |  |  |
|  | People post what they think is trendy on social media |  |  |  |
|  | People tend to exaggerate their experience for attention on social media. |  |  |  |
|  | People hide who they really are when post on social media. |  |  |  |
|  | It's easy to get a false perception of someone on social media. |  |  |  |
|  | People bend truth to be popular on social media. |  |  |  |
| Naturality |  | 3.55 | .76 | .83 |
|  | Use of filters for photos or videos is an acceptable part of social media posting. |  |  |  |
|  | Using photo filters to enhance features is perfectly fine. |  |  |  |
|  | So long as it can make me laugh, I don't mind how much preplanning, practice, and editing is behind a funny video. |  |  |  |
|  | I’m okay with staged photos as they can express the poster’s intention more clearly. |  |  |  |
|  | I’m fine with people using a lot of editing to make a post look natural. |  |  |  |
| Authenticity |  | 3.19 | .88 | .91 |
|  | For the most part, people post about their honest thoughts on social media. |  |  |  |
|  | People's social media posts reflect what they truly believe |  |  |  |
|  | People's posts portray what they really think |  |  |  |
|  | People’s genuine attitudes are reflected in their social media posts. |  |  |  |
|  | People are, in general, who they actually are on social media. |  |  |  |
|  | People tend to express their true selves on social media. |  |  |  |
| Resonance |  | 3.51 | .80 | .92 |
|  | Social media posts feel real when they resonate with me. |  |  |  |
|  | I can trust the posts that reflect my values. |  |  |  |
|  | I feel connected to a person whose social media posts resonate with my beliefs. |  |  |  |
|  | Social media feels real when other people express the same thoughts that I have. |  |  |  |
|  | I trust people whose posts resonate with my experiences. |  |  |  |
|  | Posts that I can relate to feel real to me. |  |  |  |
| Social assurance |  | 2.84 | 1.03 | .93 |
|  | I trust posts with a lot of likes. |  |  |  |
|  | Social media posts with a lot of views are trustworthy. |  |  |  |
|  | When a lot of people have positively commented on a post, it must have something real. |  |  |  |
|  | When in doubt I look at how many people liked or commented on the post. |  |  |  |
|  | In general people with a lot of followers generate trustworthy content. |  |  |  |
|  | There must be something truthful in the posts that get popular. |  |  |  |
