# Supplementary material for: A Multidimensional Approach for Evaluating Reality in Social Media: Mixed Methods Study
Source: J Med Internet Res. 2024 Aug 6;26:e52058. doi: 10.2196/52058 (PMC11336507; doi:10.2196/52058)
Supplement: Multimedia Appendix 3 [file jmir_v26i1e52058_app3.docx]

**Multimedia Appendix 3**

**Bivariate correlations between variables**

|  | 1 | 2 | 3 | 4 | 5 | 6 | 7 | 8 | 9 | 10 | 11 | 12 | 13 | 14 | 15 |
| --- | --- | --- | --- | --- | --- | --- | --- | --- | --- | --- | --- | --- | --- | --- | --- |
| 1. Falsity | — |  |  |  |  |  |  |  |  |  |  |  |  |  |  |
| 2. Authenticity | -.12* | — |  |  |  |  |  |  |  |  |  |  |  |  |  |
| 3. Naturality | .28*** | .41*** | — |  |  |  |  |  |  |  |  |  |  |  |  |
| 4. Resonance | .10^+^ | .57*** | .56*** | — |  |  |  |  |  |  |  |  |  |  |  |
| 5. Social assurance | .05 | .68*** | .54*** | .73*** | — |  |  |  |  |  |  |  |  |  |  |
| 6. SM use frequency | .26*** | .32*** | .48*** | .42*** | .46*** | — |  |  |  |  |  |  |  |  |  |
| 7. SM engagement | .17*** | .37*** | .44*** | .45*** | .40*** | .50*** | — |  |  |  |  |  |  |  |  |
| 8. Information seeking | .23*** | .27*** | .36*** | .40*** | .33*** | .40*** | .40*** | — |  |  |  |  |  |  |  |
| 9. Global realism | -.04 | .71*** | .37*** | .57*** | .65*** | .38*** | .44*** | .29*** | — |  |  |  |  |  |  |
| 10. Eudaimonic | .15** | .42*** | .42*** | .53*** | .46*** | .45*** | .46*** | .48*** | .49*** | — |  |  |  |  |  |
| 11. Hedonic | .09^+^ | .41*** | .51*** | .52*** | .38*** | .44*** | .46*** | .31*** | .47*** | .45*** | — |  |  |  |  |
| 12. Escape | .29*** | .27*** | .46*** | .42*** | .45*** | .47*** | .43*** | .37*** | .37*** | .51*** | .44*** | — |  |  |  |
| 13. Homophily | .10* | .45*** | .33*** | .46*** | .45*** | .37*** | .44*** | .31*** | .51*** | .36*** | .43*** | .32*** | — |  |  |
| 14. Surveillance | .10* | .39*** | .42*** | .52*** | .49*** | .51*** | .44*** | .45*** | .48*** | .58*** | .47*** | .49*** | .44*** | — |  |
| 15. Reflective thinking | .10* | .32*** | .24*** | .41*** | .36*** | .28*** | .38*** | .53*** | .38*** | .39*** | .24*** | .29*** | .31*** | .40*** | — |
| 16. Addictive SM use | .08 | .43*** | .38*** | .45*** | .60*** | .60*** | .43*** | .42*** | .44*** | .43*** | .33*** | .48*** | .35*** | .48*** | .35*** |

*Notes*. **p*<.05, ***p*<.01, ****p*<.001. Top five rows are factor correlations.
